# Rapid Dissemination of *bla*_NDM-5_ Gene among Carbapenem-Resistant *Escherichia coli* Isolates in a Yellow-Feather Broiler Farm via Multiple Plasmid Replicon

**DOI:** 10.3390/pathogens13050387

**Published:** 2024-05-07

**Authors:** Zhenbao Ma, Bo Wang, Dongping Zeng, Huanzhong Ding, Zhenling Zeng

**Affiliations:** 1 Guangdong Provincial Key Laboratory of Veterinary Pharmaceutics Development and Safety Evaluation, National Risk Assessment Laboratory for Antimicrobial Resistance of Animal Original Bacteria, South China Agricultural University, Guangzhou 510642, China; mazb@haid.com.cn (Z.M.); wangb@scau.edu.cn (B.W.); donytsang@scau.edu.cn (D.Z.); 2 Animal Husbandry and Fisheries Research Center of Guangdong Haid Group Co., Ltd., Guangzhou 511490, China

**Keywords:** CREC, whole-genome sequencing, horizontal transfer, genetic features

## Abstract

Although carbapenems have not been approved for animal use, carbapenem-resistant *Escherichia coli* (CREC) strains are increasingly being detected in food-producing animals, posing a significant public health risk. However, the epidemiological characteristics of CREC isolates in yellow-feather broiler farms remain unclear. We comprehensively investigated the genetic features of carbapenem-resistance genes among *E. coli* isolates recovered from a yellow-feather broiler farm in Guangdong province, China. Among the 172 isolates, 88 (51.2%) were recovered from chicken feces (88.5%, 54/61), the farm environment (51.1%, 24/47), and specimens of dead chickens (15.6%, 41/64). All CREC isolates were positive for the *bla*_NDM-5_ gene and negative for other carbapenem-resistance genes. Among 40 randomly selected isolates subjected to whole-genome sequencing, 10 belonged to distinct sequence types (STs), with ST167 (n = 12) being the most prevalent across different sources, suggesting that the dissemination of *bla*_NDM-5_ was mainly due to horizontal and clonal transmission. Plasmid analysis indicated that IncX3, IncHI2, and IncR-X1-X3 hybrid plasmids were responsible for the rapid transmission of the *bla*_NDM-5_ gene, and the genetic surrounding of *bla*_NDM-5_ contained a common mobile element of the genetic fragment designated “IS*5*-△IS*Aba125*-*bla*_NDM-5_-*ble*_MBL_-*trpF*-*dsbC*”. These findings demonstrate a critical role of multiple plasmid replicons in the dissemination of *bla*_NDM-5_ and carbapenem resistance.

## 1. Introduction

Food-producing animals are considered to serve as a significant “reservoir” of resistance genes, playing a key role in the dissemination of antibiotic resistance. New Delhi metallo-β-lactamase-1 (NDM-1) and its variants, which confer resistance to all β-lactams with the exception of aztreonam, have been globally disseminated among Gram-negative bacteria owing to rapid gene transfer between species [1,2]. Although carbapenems have not been approved for use in animals in China, a high prevalence of NDM-producing *Escherichia coli* has been observed in chicken, commercial swine, duck, and goose farms in several provinces [3,4,5,6,7].

The rapid dissemination of NDM-producing *E. coli* among food-producing animals has been boosted by the horizontal transfer of *bla*_NDM_ genes on mobile genetic elements such as insertion sequences, transposons, and their associated plasmids [8]. Insertion sequences (e.g., ISAba*125* and IS*3000*) and transposons (e.g., Tn*125*) play a vital role in the genetic variability of *E. coli* [9,10]. The IS*Aba125* insertion sequence has been reported to induce the expression of the *bla*_NDM_ gene in *Acinetobacter baumannii* [10]. Plasmids belonging to different incompatibility groups play an important role in the epidemiology of *bla*_NDM_ genes. The *bla*_NDM-5_ gene is carried by a broad range of plasmids belonging to the IncX3, IncHI2, and IncF incompatibility groups [11,12]. *E. coli* sequence types (STs) ST48, ST167, and ST10 were identified as the main epidemiological clones of *bla*_NDM-5_ associated with human infections, which pose a potential threat to public health [5,6,7]. However, the epidemiological characteristics and dissemination patterns of *bla_NDM-5_* in yellow-feather broilers remain unclear.

The cultivation area of yellow-feather broilers is mainly distributed in Southern China, including Guangdong province. It was estimated that the consumption of yellow-feather broilers reached over 825 million in Guangdong, accounting for approximately 19.7% of the overall consumption of broilers of China in 2021 (http://dara.gd.gov.cn/cxxsfx/content/ post_3803320.html, accessed on 20 June 2023). However, to our knowledge, there have been only a few reports about the relationship between carbapenem-resistant *E. coli* (CREC) isolates in healthy chicken and the environment in yellow-feather broiler farms under an “all in/all out” management strategy. One study reported the prevalence of extraintestinal pathogenic *E. coli* (ExPEC) of 22% among 926 *E. coli* isolates obtained from healthy chicken in China, including *mcr-1*-positive ExPEC isolates harboring the *bla*_NDM_ gene [3]. Another longitudinal study revealed that the prevalence of the *bla*_NDM_ gene was 13.0% among *E. coli* isolates recovered from chicken feces, and the horizontal transmission of *bla*_NDM_-carrying plasmids such as IncX3, IncA/C, and IncHI2 was observed among chicken feces and the environment niches both inside and outside of farmhouses [4]. Thus, in the present study, we investigated the prevalence and genetic features of CREC isolates obtained from different sources, including feces, tissue samples of dead chickens, and environmental samples, of a typical broilers farm under “all in/all out” management in Guangzhou City, China. We further explored the transfer mechanism of the *bla*_NDM-5_ gene among the isolates to provide a greater understanding of the dissemination mechanism, which can help to inform control strategies.

## 2. Materials and Methods

### 2.1. Farm Background

We selected a chicken farm in the Zengcheng district of Guangzhou City that had suffered from an infectious disease outbreak suspected to be caused by *E. coli* prior to our sample collection on 11 April 2018. Common antimicrobial drugs, including amoxicillin, florfenicol, doxycycline, oxytetracycline, and enrofloxacin, had been added to the feed and drinking water to prevent and treat infectious diseases for all chickens.

### 2.2. Sample Collection and CREC Isolation

A total of 172 samples, including 61 fecal samples, 64 tissue samples from dead chickens, and 47 environmental samples, were collected from the chicken farm. The environmental samples, comprising soil (n = 5), drinking water (n = 25), and feed (n = 17), and the fecal samples were collected from three chicken houses. In addition, the samples of dead chickens were obtained from different tissues to increase the isolation rate of CREC, including the lungs (n = 11), liver (n = 11), heart (n = 11), spleen (n = 11), intestine (n = 11), and brain (n = 11). All specimens were preserved in an icebox for transport to the laboratory. 

The surfaces of specimens were cleaned with sterile saline and the deep tissue was dipped using an aseptic swab in a vertical-flow clean bench to protect against environmental contamination. All samples were cultured using Luria–Bertani (LB) broth and incubated with shaking at 200 rpm and 37 °C for 14 h. CREC isolates were screened using MacConkey agar plates supplemented with 0.5 mg/L meropenem. Non-duplicate colonies were identified using 16S rRNA sequencing as reported previously [8]. The key resistance genes, including *bla*_NDM_, *bla*_KPC_, *bla*_OXA-181_, *bla*_IMP_, *mcr-1*, and *floR*, were screened using polymerase chain reaction (PCR) amplification and sequencing as previously described [13,14].

### 2.3. Antibiotic Susceptibility Testing

The minimum inhibitory concentrations (MICs) of 14 different drugs against the obtained CREC isolates, including ampicillin, cefotaxime, cefoxitin, ceftazidime, imipenem, meropenem, gentamycin, amikacin, tetracycline, tigecycline, florfenicol, fosfomycin, ciprofloxacin, and sulfamethoxazole/trimethoprim, were determined by the agar dilution method. The MIC value of colistin was determined via the broth microdilution method. The breakpoints were interpreted according to Clinical and Laboratory Standards Institute guidelines [15]. The MICs for colistin (>2 mg/L) and florfenicol (>16 mg/L) were interpreted according to clinical breakpoints or epidemiological cut-off values of the European Committee on Antimicrobial Susceptibility Testing (http://mic.eucast.org/Eucast2/, accessed on 23 May 2023). *E. coli* ATCC 25922 served as the control.

### 2.4. Whole-Genome Sequencing and Analysis

To understand the epidemiological relationship of CREC isolates obtained from different sources, the 40 isolates composed of 22 CREC from fecal samples, 8 CREC from tissue samples, and 10 CREC from environmental samples were randomly selected for whole-genome sequencing. Genomic DNA was extracted using the HiPure Bacteria DNA Kit (Magen Biotechnology Co., Ltd., Guangzhou, China) following the manufacturer’s instructions. The library was constructed and sequenced on an Illumina NovaSeq platform with a 150 bp paired-end strategy. The raw reads covered at least 100-fold of the genome; sequences of each strain and draft genomes were assembled using CLC Genomics Workbench software, version 10 (CLC Bio, Aarhus, Denmark). Multi-locus sequence types (MLSTs), antibiotic resistance genes (ARGs), plasmid types, and virulence genes of the assembled genomes were identified using Center for Genomic Epidemiology tools (https://cge.food.dtu.dk/services, accessed on 23 April 2023). 

The 40 assembled genomes were then used for core-genome alignments to produce a phylogenetic tree with Parsnp in the Harvest package (version 1.1.2). The core genome single-nucleotide polymorphism (SNP)-based phylogenetic tree was visualized and annotated using Figtree version 1.4.2. [16]. The heatmap was produced by GraphPad Prism 7.0 (GraphPad Software Inc., La Jolla, CA, USA).

### 2.5. Genetic Context of bla_NDM-5_

The genetic context of the *bla*_NDM-5_ gene of the 40 CREC strains was determined and analyzed according to the Illumina sequencing data and PCR mapping with the primers listed in Supplemental Table S1.

### 2.6. Plasmid Sequencing and Annotation

The non-IncX3 *bla*_NDM-5_-carrying plasmids designated p8C57-NDM and p8C59-NDM from strains GZB8C57M and GZB8C59M, respectively, were selected and sequenced by the long-read MinION sequencer (Nanopore, Oxford, UK). The two sequences were then assembled and calibrated according to the Illumina sequencing data using unicycler 0.4.3. The two complete genomes of the plasmids were annotated and analyzed using the RAST (https://rast.nmpdr.org/rast.cgi, accessed on 1 February 2023), ISfinder (https://www-is.biotoul.fr/, accessed on 1 February 2023), ResFinder (https://cge.food.dtu.dk/services/ResFinder/, accessed on 1 February 2023), BRIG (http://sourceforge.net/projects/brig/, accessed on 12 March 2023), and Vector NTI (Invitrogen) programs.

### 2.7. Conjugation Assay

To determine the transferable ability of the *bla*_NDM_-positive plasmids of 10 distinct *E. coli* sequence types (STs), a conjugation experiment was performed by the broth-mating method with sodium azide-resistant *E. coli* J53 serving as the recipient strain. Overnight culture of donor strains and *E. coli* J53 were mixed at a ratio of 1:1 in LB broth and then incubated overnight at 37 °C. The transconjugants were selected on MacConkey agar plates supplemented with meropenem (0.5 mg/L) and sodium azide (200 mg/L).

### 2.8. Nucleotide Sequence Accession Numbers

The 40 assembled genomes of *bla*_NDM-5_-positive *E. coli* have been deposited in GenBank under the BioProject with accession number PRJNA628022. The nucleotide sequences of plasmids p8C57-NDM and p8C59-NDM have been deposited in the GenBank database under accession numbers MT407546 and MT407547, respectively.

## 3. Results

### 3.1. Prevalence of CREC Isolates and Drug Susceptibility

**Figure 1:**
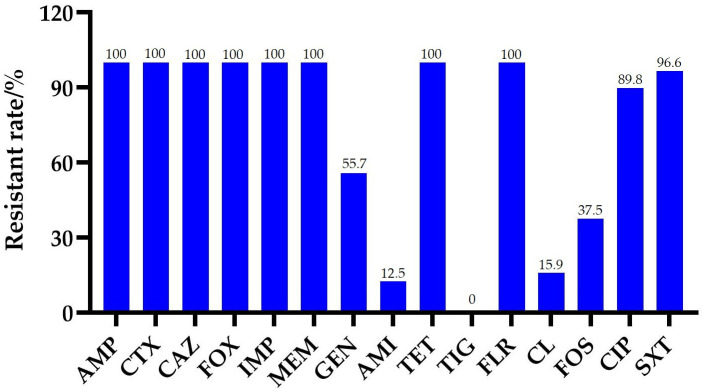
Antibiotic resistance rates of 88 CREC isolates. Abbreviations: AMP, ampicillin; CTX, cefotaxime; CAZ, ceftazidime; FOX, cefoxitin; IMP, imipenem; MEM, meropenem; GEN, gentamycin; AMI, amikacin; TET, tetracycline; TIG, tigecycline; FLR, florfenicol; FOS, fosfomycin; CIP, ciprofloxacin; SXT, sulfamethoxazole/trimethoprim.

A total of 88 CREC strains were isolated from 172 (51.2%) samples in the chicken farm, including feces (n = 54), the intestine (n = 6), heart (n = 1), liver (n = 1), spleen (n = 1), lung (n = 1), soil (n = 5), drinking water (n = 16), and feed (n = 3). Thus, the isolation rate of CREC isolates from fecal, tissue, and environmental samples was 88.5%, 15.6%, and 51.1%, respectively. Antibiotic susceptibility testing showed that all CREC isolates were resistant to all β-lactams, tetracycline, and florfenicol, and no strain exhibited tigecycline resistance. The resistance rates of other antibiotics were as follows: 96.6% for sulfamethoxazole/trimethoprim, 89.8% for ciprofloxacin, 55.7% for gentamycin, 37.5% for fosfomycin, 15.9% for colistin, and 12.5% for amikacin (see Figure 1). PCR amplification showed that all 88 CREC isolates were positive for the *bla*_NDM-5_ gene and negative for other carbapenemase genes. Moreover, the detection rate of *mcr-1* and *floR* was 15.9% (14/88) and 94.3% (83/88) in these CREC isolates, respectively.

### 3.2. Whole-Genome Sequencing Analysis

**Figure 2:**
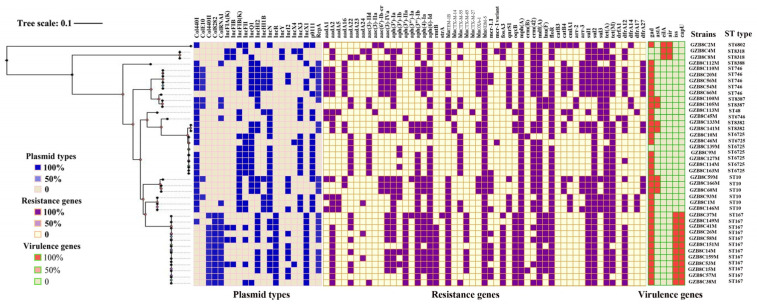
Heatmap representation of sequenced data for plasmid types, antibiotic resistance genes, virulence genes, and multi-locus sequence types of 40 *bla*_NDM-5_-positive *E. coli* isolates. The single-nucleotide polymorphism-based phylogenetic tree was created by Parsnp in the Harvest package (version 1.1.2), and was visualized and annotated using Fig tree version 1.4.2.

To understand the epidemiological relationship of CREC isolates from different sources, 40 CREC isolates were randomly selected for whole-genome sequencing. MLST results indicated that the 40 *bla*_NDM-5_-positive *E. coli* isolates were divided into 10 distinct STs, with ST167 (n = 12) being the most prevalent, followed by ST6725 (n = 7), ST10 (n = 6), ST746 (n = 6), ST8318 (n = 2), ST8382 (n = 2), and ST8387 (n = 2); the remaining STs were identified in one isolate each (Figure 2). A core genome SNP-based phylogenetic analysis further confirmed the generality of CREC isolates with the same STs. It is noteworthy that several STs such as ST10, ST167, ST6725, and ST746 were distributed in various sources, including feces, drinking water, and feed, which implied the clonal spread and circulation of NDM-producing *E. coli* isolates in this chicken farm. Moreover, the 40 CREC isolates were found to harbor a variety of plasmid replicon types, including IncHI2, IncF, and IncX, and diverse ARGs conferring resistance to important clinical antibiotics for human and veterinary medicine, such as β-lactams (*bla*_TEM-1B_, *bla*_CTX-M-14_, *bla*_CTX-M-27_, *bla*_CTX-M-55_, *bla*_CTX-M-65_, and *bla*_OXA-1_), aminoglycosides [*aac(6′)-Ib-cr* and *rmtB*], fosfomycin (*fosA3*), and florfenicol (*floR*) (Figure 2). Unlike the diversity of ARGs, only five different type of virulence genes were detected in these strains. The *gad* gene, with glutamate decarboxylase function, was the most prevalent virulence gene identified (Figure 2). The conjugation assay demonstrated that all 40 CREC isolates except for ST167 were successfully transferred in the *bla*_NDM-5_-positive transconjugants (Supplemental Table S2).

### 3.3. Genetic Surrounding of the bla_NDM-5_ Gene

**Figure 3:**
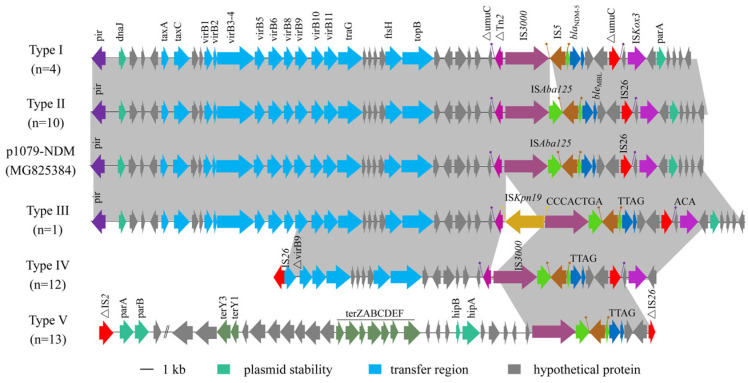
Linear comparison of the genetic structures of the *bla*_NDM-5_ gene. Arrows indicate the positions and directions of gene transcription. Regions with >99% homology are shaded in gray. △ indicates a truncated gene.

The length of the 40 *bla*_NDM_-bearing contigs ranged from 3414 to 108,589 bp. PCR mapping and sequencing indicated five genetic structures for these isolates, designated type I (n = 4), type II (n = 10), type III (n = 1), type IV (n = 12), and type V (n = 13) (Figure 3). Structure types I-III of *bla*_NDM-5_ possessed an entire IncX3 plasmid backbone, which was almost identical to that of the IncX3 plasmid p1079-NDM (GenBank accession no. MG825384). The type IV genetic structure (~24.0 kb) contained a variant region of the *bla*_NDM-5_ gene and a partial IncX3 plasmid backbone, which showed 100% homology with the corresponding region of the IncX3 plasmid p1079-NDM. In the type IV genetic structure, the type IV secretion system associated with *virB9* was truncated by an insertion sequence (IS*26*), which may lead to the loss of the conjugative transfer region of the IncX3 plasmid. In contrast to the other genetic structure types, the complete sequence of the type V genetic structure (~108 kb) included a *bla*_NDM-5_-carrying variant region, plasmid partitioning system *(parA-parB*), and a set of tellurite resistance determinants (terYZABCDEF), which exhibited >99% identity to the corresponding region of the IncHI2 plasmid pHNYJC8 (GenBank accession no. KY019259). In addition, the conserved region IS*5*-△IS*Aba125*-*bla*_NDM-5_-*ble*_MBL_-*trpF*-*dsbC*-IS*26* was found in all genetic structures (Figure 3). The genetic context of *bla*_NDM-5_ had a deletion of △IS*Aba125* located downstream of IS*5* in the type I genetic structure. The insertion sequence IS*Kpn19* was inserted upstream of IS*3000* with an 8 bp CCCACTGA direct repeat in type III. This result suggested that the variant region was distinct, which may be due to the insertion, recombination, and/or deletion of a mobile element. In addition, the conjugation assay demonstrated that all 40 CREC isolates except for ST167 were successfully obtained in the *bla*_NDM-5_-positive transconjugants (Supplemental Table S2). The genetic structure types I-III were classified as IncX3 plasmids; however, the genetic features of plasmids carrying types IV and V genetic structures were unclear. Thus, the plasmids carrying type IV and type V genetic structures were selected for further analysis to reveal the genetic features using nanopore sequencing. 

### 3.4. Analysis of Plasmids p8C57-NDM and p8C59-NDM

Two plasmids with a type IV and type V genetic structure, p8C57-NDM and p8C59-NDM, respectively, were selected for complete sequencing using the long-read MinION sequencer.

**Figure 4:**
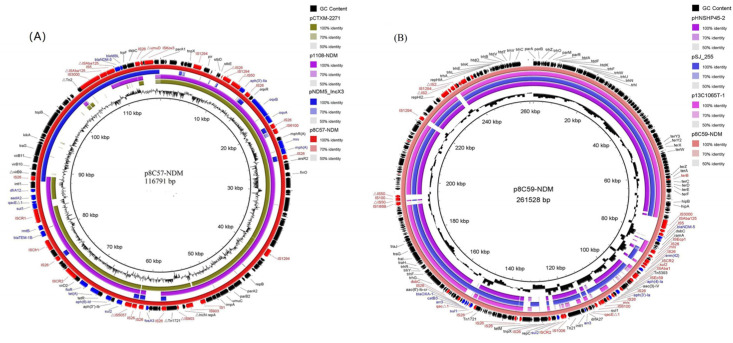
Ring comparison of plasmids p8C57-NDM (**A**) and p8C59-NDM (**B**) constructed using BRIG. Blue arrows indicate resistance genes, red arrows indicate insertion sequences, and black arrows without annotation indicate hypothetical proteins. The sequences of plasmids pHNSHP45-2 (KU341381), p13C1065T-1 (CP019260), pSJ_255 (CP011062), p1108-NDM (MG825381), pCTXM-2271 (MF589339), and pNDM5_IncX3 (KU761328) were downloaded from the National Center for Biotechnology Information nucleotide database.

The complete length of plasmid p8C57-NDM was 116,791 bp, showing >99% sequence identity with 79% coverage to plasmid p1108-NDM (GenBank accession no. MG825381) of *E. coli* collected from a raw chicken product in China and >99% identity with 77% coverage to plasmid pCTXM-2271 (GenBank accession no. MF589339) of *E. coli* strain 2271. Plasmid p8C57-NDM was classified as an IncR-X1-X3 hybrid plasmid according to the four main plasmid backbone regions. The first segment contained a ~24.0 kb genetic structure (△*virB9*-*virB10*-*bla*_NDM-5_-IS*Kox3*-*hp*), which exhibited 100% nucleotide identity with p1079-NDM (GenBank accession no. MG825384). The second backbone fragment (~9.3 kb) contained an IncX1 plasmid replication region and addiction system (*parA*-*parG*-*ddp3*-△*repX*-IS*1294*-△*repX*-*pir*-s*tbD/E*), which showed >99% identity to the corresponding region of plasmid p14EC0176 (GenBank accession no. CP024136) and the IncX1 plasmid pOLA52 (EU370913). However, plasmid pOLA52 lacked the IncX plasmid initiator replication protein RepX and IS1294. The third fragment (*arsR2*-*finO*-*vagC/D*-*pifC*) displayed >99% identity to the corresponding region of the archetypal IncI1 plasmid R64 (GenBank accession no. AP005147) backbone. It is possible that the IncI1 segment is captured by IS*1294* upstream of *pifC*. The fragment IS*1294* downstream was linked with the fourth genetic structure (*resD*-*repB*-*parA*-*parB*-*impA*-IS*1*-*repA*), which showed >99% nucleotide identity with the IncR plasmid pKP1780 (GenBank accession no. JX424614) and encoded the IncR replication initiation protein RepB, plasmid partitioning protein ParA-ParB, and IncN replication initiation protein RepA (Figure 4A). In addition, plasmid p8C57-NDM carried multiple mosaic multi-resistance regions (MRRs) consisting of various resistance genes, including *aadA2*, *aph(3”)-Ib*, *aph(3′)-IIa*, *aph(6)-Id*, *rmtB*, *bla*_TEM-1B_, *bla*_NDM-5_, *oqxAB*, *fosA3*, *floR*, *mph(A*), *tet(A)*, *sul1*, *sul2*, and *dfrA12*, along with complete or truncated insertion sequences and transposons (such asIS*26*, IS*1294*, IS*50*, IS*6100*, IS*1*, IS*903*, IS*5075*, IS*CR1*, IS*CR2*, IS*Cfr1*, Tn*2*, and Tn*1721*). A total of 10 copies of IS*26* were distributed in plasmid p8C57-NDM, mainly in MRRs. These findings suggested that IS*26* might have played a key role in the formation of plasmid p8C57-NDM via transposition and homologous recombination. Plasmid p8C57-NDM lacks an entire transferable region, which could explain why the *bla*_NDM-5_-carrying plasmid p8C57-NDM failed to transfer to the recipient *E. coli* J53.

Plasmid p8C59-NDM was 261,528 bp in size and belonged to IncHI2/ST3. The backbone region, corresponding to plasmid replication, conjugative transfer, maintenance, and stability, was almost identical to that of other IncHI2 plasmids such as pHNSHP45-2 (GenBank accession no. KU341381) of *E. coli* strain SHP45 recovered from a pig in Shanghai, China; p13C1065T-1 (GenBank accession no. CP019260) from *E. coli* of chicken originated from Hong Kong; and pSJ_255 (GenBank accession no. CP011062) of *E. coli* str. Sanji collected from the duodenum of a pheasant in China (Figure 4B). However, the variant region (62,118 bp) of plasmid p8C59-NDM was distinct, which was found to carry 14 different resistance genes such as *tet(M)*, *aac(6′)-Ib-cr*, *fosA3*, *bla*_OXA-1_, *bla*_NDM-5_ and *arr-3* (Figure 4B). 

## 4. Discussion

Multidrug-resistant (MDR) *E. coli* has become a worrisome issue that poses a threat to public health and serves as a major reservoir of antibiotic resistance genes that may be responsible for treatment failure events in human clinical and veterinary medicine [17]. The emergence and spread of carbapenem-resistant *Enterobacteriaceae* (CRE) have caused a variety of infectious diseases associated with high mortality rates, posing a critical threat to clinical treatment. A longitudinal study found that 13.0% of *E. coli* strains recovered from chicken feces carried the *bla*_NDM_ gene in 2015–2016, and 55.8% of fecal and environmental samples were found to harbor *bla*_NDM_-positive bacteria during the breeding periods of 2017–2021, indicating the persistence of *bla*_NDM_-positive bacteria in chickens and farm environments. This phenomenon is likely due to contamination by exogenous materials and the spread of *bla*_NDM_-positive bacteria within the broiler farm system [4]. Consistent with this previous research, a recent study demonstrated that CRE-negative 1-day-old broilers acquired *bla*_NDM_ within 24 h of transfer, with a detection rate reaching up to 18.6% [18]; this same study suggested that the contaminated in-house environment contributes to the persistence and transmission of *bla*_NDM_-bearing bacteria in Chinese poultry farms. In the present study, we found that the carriage rate of *bla*_NDM-5_-positive isolates recovered from fecal samples and environmental samples in the yellow-feather broiler chicken farm in Guangdong was 88.5% and 51.1%, respectively, which was significantly higher than the rates previously reported [4,19,20]. Drugs such as ampicillin and amoxicillin can enrich both CRE and antibiotic-susceptible *E. coli* in the gastrointestinal tract, consequently promoting the transmission of *bla*_NDM_-bearing plasmids in the gut microbiome [21]. Therefore, the high occurrence of *bla*_NDM_ in the chicken farm may be caused by the use of amoxicillin. Notably, these CREC strains exhibited resistance to tetracycline and florfenicol, which could result from co-selection with other drugs even without the selective pressure of β-lactams. In addition, 15.9% of the CREC isolates in this chicken farm exhibited a colistin-resistance pattern, and these isolates carried the *mcr-1* gene. Compared with the reported 23.0–26.9% prevalence of *mcr-1* among CREC isolates from chicken farms [18,20], the detection rate of *mcr-1* was lower in this study, which may be due to the ban of colistin as a feed additive for animals in China [22].

ST167, ST10, ST6725, and ST746 were the most common identified ST clones of the *bla_NDM_* gene. The most widespread STs were determined to be ST167, ST10, ST48, ST746, ST617, and ST410, which have been identified in humans, pigs, chickens, and other sources [23]. However, ST6725 was only isolated from the poultry farm. It is noteworthy that ST10, ST167, ST6725, and ST746 were distributed among various sources, including feces, drinking water, and feed, which implied the clonal spread and circulation of NDM-producing *E. coli* isolates in this chicken farm. Environmental sources such as sewage, soil, and feed serve as important carriers in the spread of antibiotic resistance genes such as extended-spectrum β-lactamase (ESBL) and *mcr-1* genes [5,24]. Samples of water and feed, which are necessary for chicken growth, were collected from the chicken house for analysis; thus, chickens were exposed to CREC isolates through contamination of their regular drinking water and feed, which could be responsible for causing infections. In addition, ST10 and ST746 were identified in the tissue samples of dead chicken, feces, and environment samples, suggesting that chicken colibacillosis can be caused by *E. coli* isolates recovered from the environmental reservoir, supporting the viewpoint of Poirel et al. [17]. Notably, ST167, ST10, and ST48 belonged to clone complex CC10, which is closely related to the infectious disease of humans and is responsible for the transmission of multiple ARGs, including *bla*_NDM_ [18,25,26,27]. These results revealed that the yellow-feather broiler chicken and its environment are an important reservoir for NDM-positive *E. coli* and pose a potential threat to public health. Previous studies have indicated the presence of *E. coli* isolates carrying *mcr-1*-*bla*_NDM-5_ among healthy people, poultry production, and swine in various regions of China, and several *E. coli* isolates of different STs such as ST167 and ST10 have been identified to be responsible for the spread and diffusion of *mcr-1* and *bla*_NDM-5_ [28,29]. In this study, IncI2 and IncX4 bearing the *mcr-1* gene were detected in multiple *E. coli* STs, including ST167 and ST10, which have a close association with clinical infection and thus warrant further attention (see Supplemental Figure S1).

Mobile genetic elements such as insertion sequences and plasmids are primarily responsible for the spread of *bla*_NDM_. The IncX3 plasmid is considered to be the main carrier of the *bla*_NDM_ gene and has been shown to mediate the horizontal transmission of the *bla*_NDM_ gene in poultry farms [18,19]. More recently, IncHI2 plasmids have also been identified as key vectors contributing to the rapid transmission of the *bla*_NDM_ gene [4,30]. The genetic context of the *bla*_NDM_ gene may explain these patterns, possessing a conserved structure (IS*Aba125*-*bla*_NDM-5_-*ble*_MBL_-*trpF*-*dsbC*) but flanked by multiple types of insertion sequences on either one or both sides. Indeed, the results of this study demonstrated that insertion sequences such as IS*26*, IS*3000*, IS*Kpn19,* and IS*Aba125* play an important role in the recombination of the *bla_NDM_* gene between different genetic environments. In this study, the *bla*_NDM-5_ gene was located on the plasmids IncX3, IncR-X1-X3, and IncHI2 of the CREC isolates. Furthermore, the genetic context of *bla*_NDM-5_ was found to be quite similar in these three plasmids, suggesting that the *bla*_NDM-5_ gene in the IncR-X1-X3 hybrid and IncHI2 plasmids may have originated from the IncX3 plasmid by the recombination or transposition of IS*26* and IS*3000*. Plasmid p8C57-NDM contained a ~24.0 kb IncX3 genetic structure (△*virB9*-*virB10*-*bla*_NDM-5_-IS*Kox3*-*hp*) and the *virB9* gene was truncated by IS*26*, which supports this hypothesis. In addition, plasmid p8C57-NDM was similar to a reported *bla*_NDM-5_-bearing IncR-IncN-IncX1 hybrid plasmid with 93% coverage and 99.9% identity, which was identified in the *E. coli* ST1771 strain collected from broiler chicken farms in Anhui province. This suggests that the hybrid plasmid may have been disseminated and evolved independently in multiple geographical locations in China. Interestingly, plasmid p8C57-NDM was limited to the *E. coli* ST167 strains identified in this study due to it lacking the transferability-associated region in accordance with the structure of the *bla*_NDM-5_-bearing IncR-IncN-IncX1 hybrid plasmid. The *bla*_NDM-5_-bearing IncX3 plasmid found in 15 *E. coli* strains showed three different genetic structures, which may be caused by the recombination of insertion sequences such as IS*Kpn19* and IS*Aba125*, consistent with the *bla*_NDM-5_-carrying IncX3 plasmid found in CRE strains isolated from retail meat in Guangzhou, China [31]. The *bla*_NDM-5_-bearing IncHI2 plasmid was previously detected in *E. coli* recovered from ducks, pigs, and fish in Guangdong province, China [30,32]. Plasmid p8C59-NDM belongs to IncHI2/ST3 with a typical backbone region, which exhibited extremely high identity (>99.9%) to the IncHI2 plasmid pHNSHP45-2 (GenBank accession no. KU341381), p13C1065T-1 (GenBank accession no. CP019260), and the *bla*_NDM-5_-bearing IncHI2 plasmids such as GDQ8D151_plasmid1 (duck), pHNBYF33-1 (fish), and pHNGD64-NDM (pig). This result implied that the *bla*_NDM-5_-carrying IncHI2 plasmid may have rapidly spread from food-producing animals in Southern China, which warrants attention and further monitoring for the sake of public health and control. 

Notably, this work represents only a snapshot in time related to the rapid dissemination of CREC isolates in a yellow-feather chicken farm; thus, further longitudinal research is warranted. We identified a high carriage rate of *bla*_NDM_-positive strains recovered from various sources; the IncX3, IncR-X1-X3 hybrid, and IncHI2 plasmids were the main carrier of the *bla*_NDM-5_ gene and largely responsible for its rapid dissemination in the chicken farm. Our findings suggest that the *bla*_NDM-5_ gene in the IncR-X1-X3 hybrid plasmid and IncHI2 plasmids may have originated from the IncX3 plasmid by the recombination or transposition of IS*26* and IS*3000*. However, we do not have sufficient evidence to explain the persistence and transmission mechanism of the *bla*_NDM_ gene. Thus, in future studies, we plan to collect a wider range of sample types from multiple farms in diverse geographic locations to gain a better understanding of the persistence and spread of the *bla*_NDM-5_ gene.

## 5. Conclusions

In summary, we comprehensively analyzed the prevalence of *bla*_NDM-5_-bearing *E. coli* isolates from diverse sources of a chicken farm, including specimen samples of dead chickens, fecal samples, drinking water, and feed. The results implied that *bla*_NDM-5_-bearing *E. coli* strains are widely circulating among different sources in this chicken farm. The high prevalence of CREC isolates in this farm poses a potential risk to animal health due to the limited drug treatment for *E. coli* infections, warranting further surveillance. To our best knowledge, this is the first report of the *bla*_NDM-5_-bearing IncR-X1-X3 hybrid plasmid in a chicken farm.

## Data Availability

The original contributions presented in the study are included in the article and Supplementary Materials.

## Supplementary Materials

The following supporting information can be downloaded at https://www.mdpi.com/article/10.3390/pathogens13050387/s1, Figure S1: Liner comparison of the *mcr-1*-carrying plasmids; Table S1: Primers of PCR mapping; Table S2: Characterization of 40 *bla*_NDM-5_-positive *E. coli* isolates from various sources in the chicken farm.
