# Supplementary material for: Rapid Dissemination of blaNDM-5 Gene among Carbapenem-Resistant Escherichia coli Isolates in a Yellow-Feather Broiler Farm via Multiple Plasmid Replicon
Source: Pathogens. 2024 May 7;13(5):387. doi: 10.3390/pathogens13050387 (PMC11124502; doi:10.3390/pathogens13050387)
Supplement: Supplementary file 1 [file pathogens-13-00387-s001.zip › pathogens-2944535-supplementary.pdf]

Table S1 Primers of PCR mapping

Name	Prime Sequence (5' to 3')	Target fragment
P1	F:GCGTAGCGTTTCCATAGCGG<br>R:GTTGACGGGTCTTTGGTGCT	1912 bp
P2	F:CCAATGCCATCGTTTACTTC<br>R:CGCTTATTCGCACCTTCCCT	1428 bp
P3	F:GGTGACAGGCAGTGTAAGAG<br>R:TTTGAAATAGCCCAGCAGTA	1251 bp
P4	F:CAGAATGAACATCCGCACCT<br>R:CCCGTCCACAATAGCAAAGT	1756 bp
P5	F:TGAAATGGCATGGGAATGAG<br>R:TTTCTGCGACAGTGATAGCG	1294 bp

Note: F: forward primer; R: reverse primer.

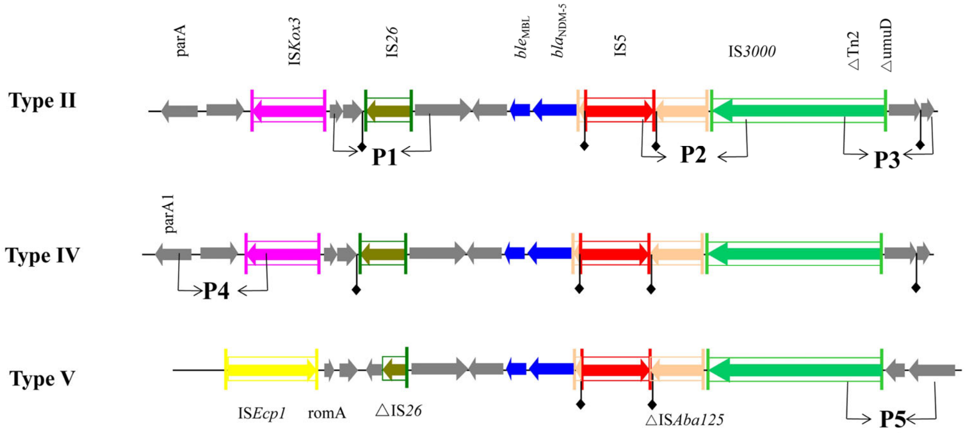

Figure S1. Liner comparison of the *mcr-1*-carrying plasmids. Arrows indicate the positions of the genes and the direction. Regions with > 99% homology are shaded in gray. (A) IncI2 plasmid. (B) IncX4 plasmid.

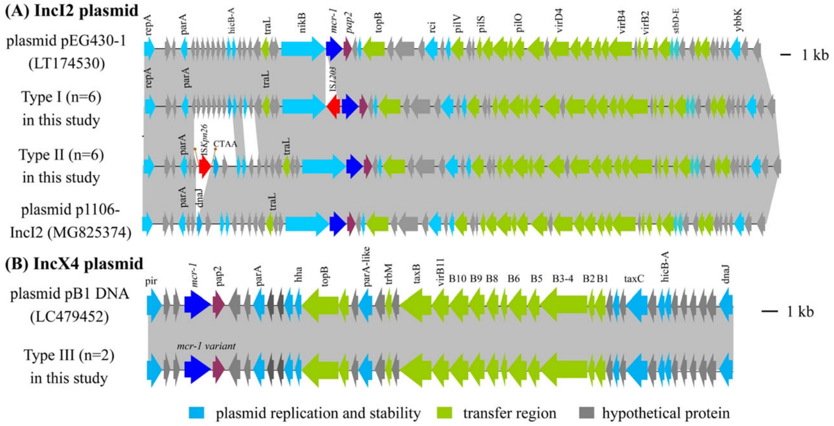

Table S2 Characterization of 40 *bla*$_{NDM-5}$-positive *E. coli* isolates from various sources in the chicken farm

Strain	Source	MLST	Surrounding regions of *bla*$_{NDM-5}$	Location of *mcr-1* and *mcr-1* variant	Other resistance phenotype [a]	Transconjugants [b]
GZB8C1M	feces	ST10	Type II	-	TET/FLR/CIP/SXT	+
GZB8C2M	feces	ST6802	Type II	-	GEN/TET/FLR/CIP	+
GZB8C4M	feces	ST8318	Type I	*mcr-1*/IncX4	GEN/TET/FLR/CL/FOS/CIP/SXT	+
GZB8C8M	feces	ST8318	Type I	-	GEN/TET/FLR/FOS/CIP/SXT	+
GZB8C9M	feces	ST6725	Type II	-	GEN/TET/FLR/FOS/CIP/SXT	+
GZB8C10M	feces	ST6725	Type II	-	GEN/TET/FLR/FOS/CIP/SXT	+
GZB8C14M	feces	ST167	Type IV	*mcr-1*/IncI2	GEN/AMI/TET/FLR/CL/FOS/CIP/SXT	-
GZB8C15M	feces	ST167	Type IV	*mcr-1*/IncI2	GEN/AMI/TET/FLR/CL/FOS/CIP/SXT	-
GZB8C20M	feces	ST746	Type V	*mcr-1*/IncI2	GEN/TET/FLR/CL/FOS/CIP/SXT	+
GZB8C26M	feces	ST167	Type IV	*mcr-1*/IncI2	GEN/AMI/TET/FLR/CL/FOS/CIP/SXT	-
GZB8C37M	feces	ST167	Type IV	-	TET/FLR/CIP/SXT	-
GZB8C38M	feces	ST167	Type IV	-	GEN/AMI/TET/FLR/FOS/CIP/SXT	-
GZB8C41M	feces	ST167	Type IV	-	GEN/AMI/TET/FLR/FOS/CIP/SXT	-
GZB8C45M	feces	ST6746	Type II	-	GEN/TET/FLR/CIP/SXT	+
GZB8C46M	feces	ST6725	Type II	*mcr-1*/IncX4	GEN/TET/FLR/CL/CIP/SXT	+
GZB8C53M	feces	ST167	Type IV	-	GEN/AMI/TET/FLR/FOS/CIP/SXT	-
GZB8C54M	feces	ST746	Type V	-	GEN/TET/FLR/CIP/SXT	+
GZB8C56M	feces	ST746	Type V	*mcr-1*/IncI2	GEN/TET/FLR/CL/CIP/SXT	+
GZB8C57M	feces	ST167	Type IV	*mcr-1*/IncI2	GEN/AMI/TET/FLR/CL/FOS/CIP/SXT	-
GZB8C58M	feces	ST167	Type IV	-	GEN/AMI/TET/FLR/FOS/CIP/SXT	-
GZB8C59M	feces	ST10	Type V	*mcr-1*/IncI2	TET/FLR/CL//FOS/SXT	+
GZB8C60M	feces	ST10	Type V	*mcr-1*/IncI2	TET/FLR/CL//FOS/SXT	+

GZB8C66M	heart	ST746	Type V	-	GEN/TET/FLR/SXT	+
GZB8C93M	spleen	ST10	Type I	-	TET/FLR/CIP/SXT	+
GZB8C100M	lung	ST8387	Type V	-	GEN/TET/FLR/FOS/CIP/SXT	+
GZB8C105M	intestinal	ST8387	Type II	-	GEN/TET/FLR/FOS/CIP/SXT	+
GZB8C110M	intestinal	ST746	Type V	-	TET/FLR/FOS/CIP/SXT	+
GZB8C112M	intestinal	ST8388	Type V	-	GEN/TET/FLR/SXT	+
GZB8C113M	intestinal	ST48	Type I	-	GEN/TET/FLR/CIP/SXT	+
GZB8C114M	intestinal	ST6725	Type II	-	GEN/TET/FLR/CIP/SXT	+
GZB8C127M	soil	ST6725	Type II	-	TET/FLR/CIP/SXT	+
GZB8C133M	drinking water	ST8382	Type V	-	TET/FLR/FOS/CIP/SXT	+
GZB8C139M	drinking water	ST6725	Type II	-	GEN/TET/FLR/FOS/CIP/SXT	+
GZB8C141M	drinking water	ST8382	Type V	-	TET/FLR/FOS/CIP/SXT	+
GZB8C146M	drinking water	ST10	Type III	*mcr-1*/IncI2	TET/FLR/CL/CIP/SXT	+
GZB8C149M	drinking water	ST167	Type IV	-	GEN/AMI/TET/FLR/FOS/CIP/SXT	-
GZB8C151M	drinking water	ST167	Type IV	*mcr-1*/IncI2	GEN/AMI/TET/FLR/CL/FOS/CIP/SXT	-
GZB8C159M	feed	ST167	Type IV	*mcr-1*/IncI2	GEN/AMI/TET/FLR/CL/FOS/CIP/SXT	-
GZB8C163M	feed	ST6725	Type II	-	GEN/TET/FLR/CIP/SXT	+
GZB8C166M	feed	ST10	Type V	*mcr-1*/IncI2	GEN/TET/FLR/CL/FOS/SXT	+

Note: a: GEN, gentamycin; AMI, amikacin; TET, tigercycline; FLR, florfenicol; CL, colistin; FOS, fosfomycin; CIP, ciprofloxacin; SXT, sulfamethoxazole/trimethoprim.

b: Transconjugants: “+”, Detect transconjugants; “-”,No detect.
